# Supplementary material for: TMPRSS2 and furin are both essential for proteolytic activation of SARS-CoV-2 in human airway cells
Source: Life Sci Alliance. 2020 Jul 23;3(9):e202000786. doi: 10.26508/lsa.202000786 (PMC7383062; doi:10.26508/lsa.202000786)
Supplement: Supplementary file 1 [file LSA-2020-00786_SdataF2_F3.pptx]

## Slide 1
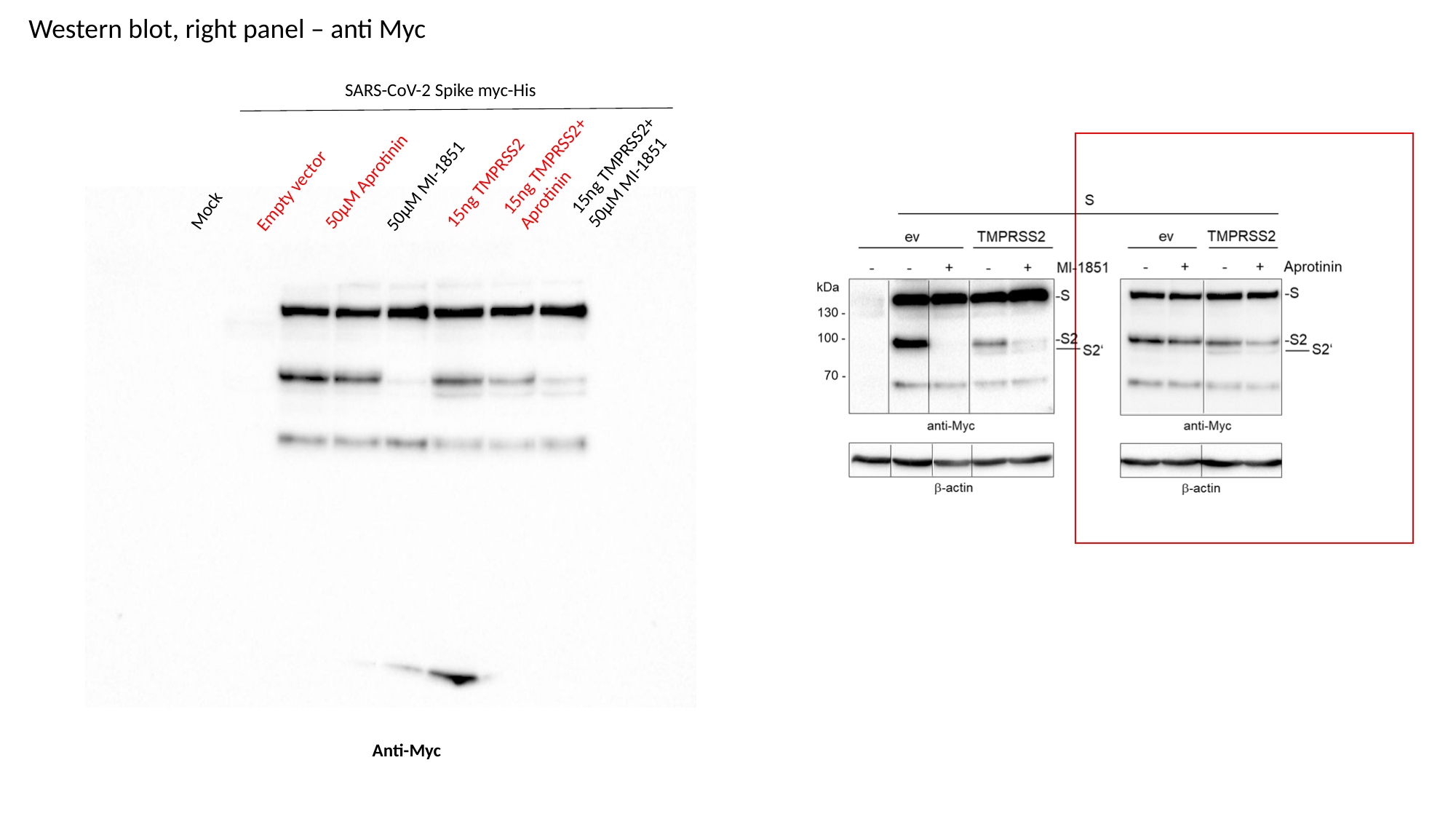

Western blot, right panel – anti Myc
SARS-CoV-2 Spike myc-His
15ng TMPRSS2+
50µM MI-1851
15ng TMPRSS2+
Aprotinin
15ng TMPRSS2
50µM MI-1851
50µM Aprotinin
Empty vector
Mock
Anti-Myc

## Slide 2
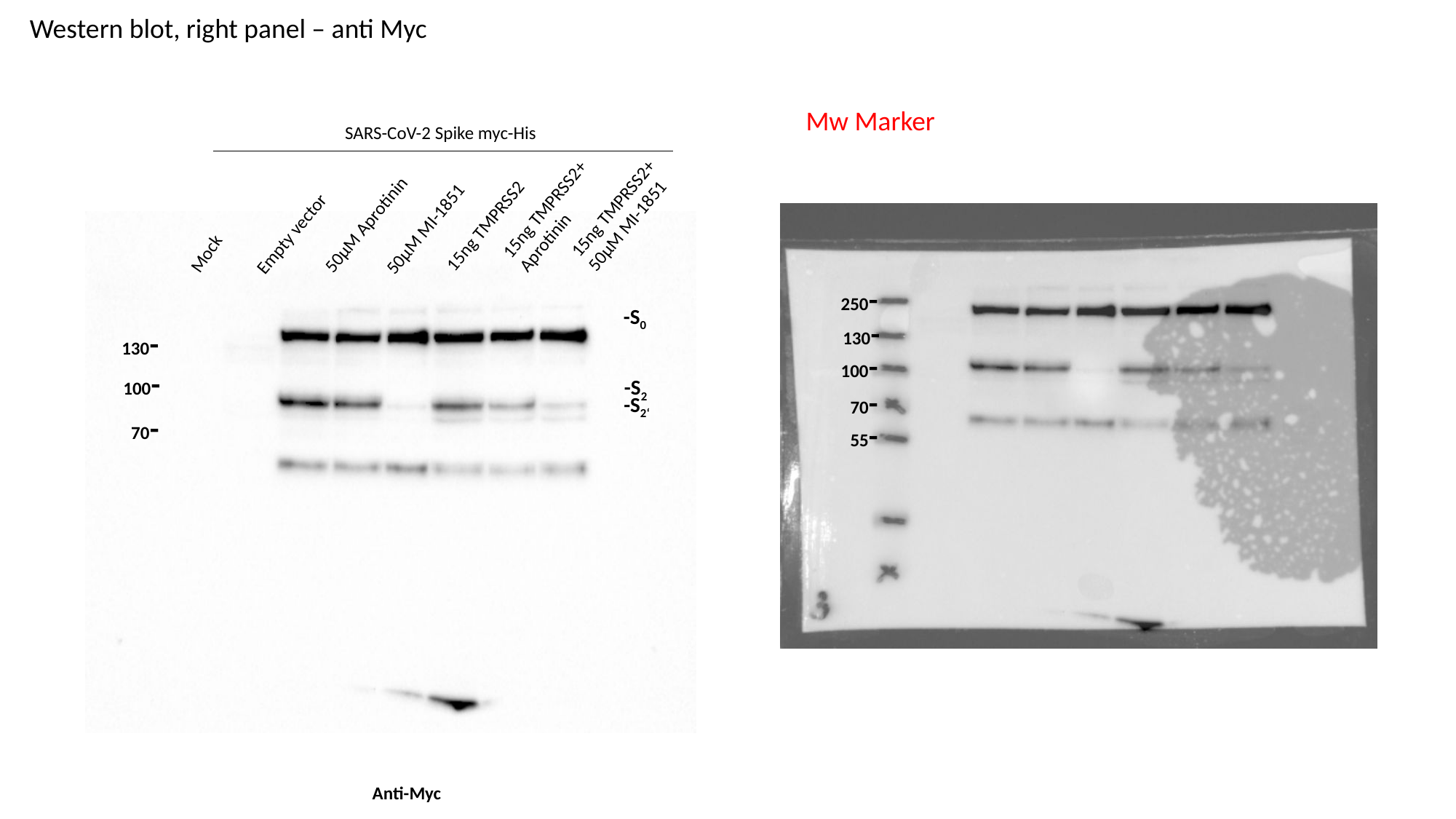

Western blot, right panel – anti Myc
Mw Marker
SARS-CoV-2 Spike myc-His
15ng TMPRSS2+
50µM MI-1851
15ng TMPRSS2+
Aprotinin
15ng TMPRSS2
50µM MI-1851
50µM Aprotinin
Empty vector
Mock
250-
-S0
130-
130-
100-
100-
-S2
70-
-S2‘
70-
55-
Anti-Myc

## Slide 3
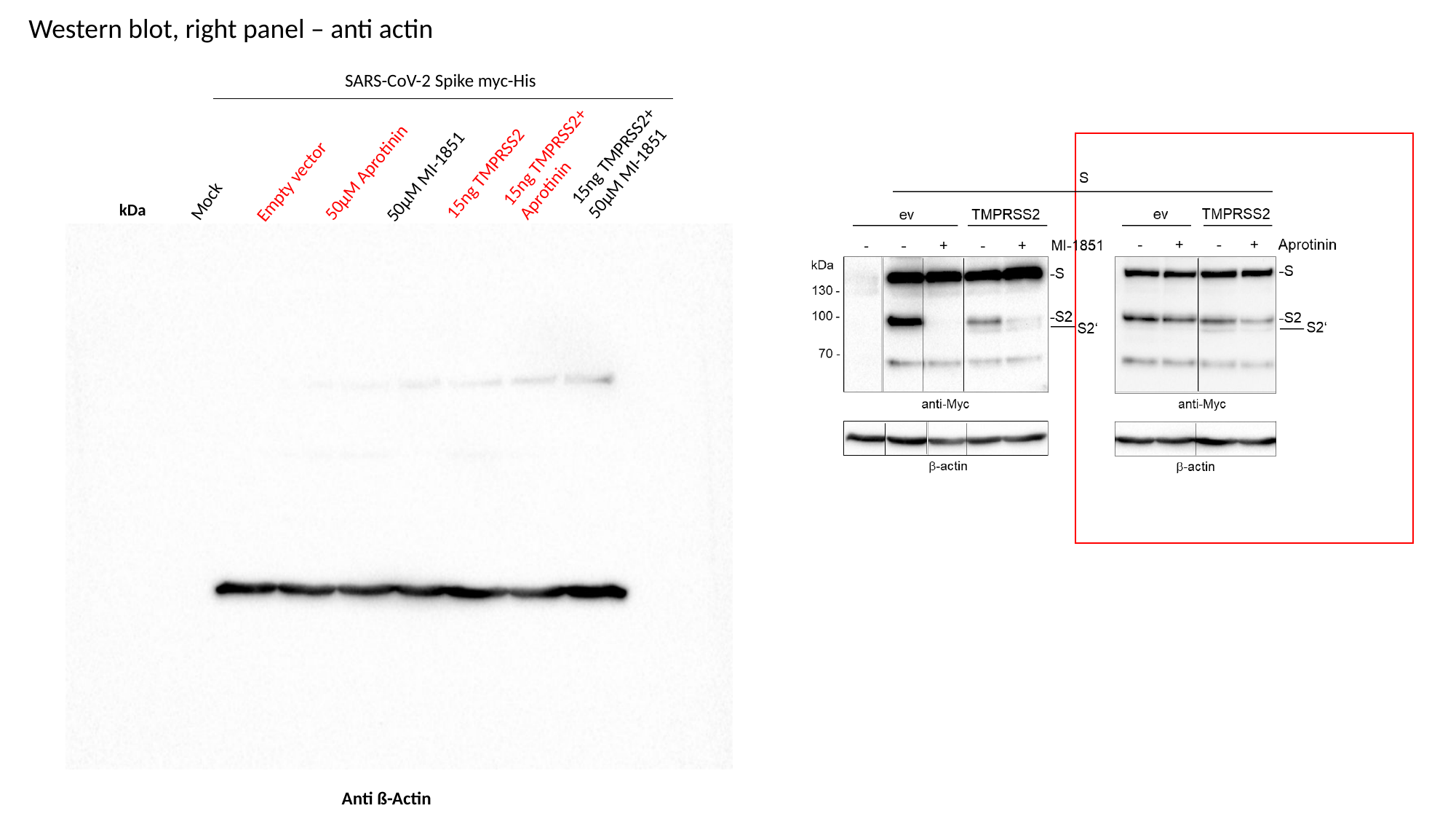

Western blot, right panel – anti actin
SARS-CoV-2 Spike myc-His
15ng TMPRSS2+
50µM MI-1851
15ng TMPRSS2+
Aprotinin
15ng TMPRSS2
50µM MI-1851
50µM Aprotinin
Empty vector
Mock
kDa
250-
130-
100-
-S2
-S2‘
70-
55-
Anti ß-Actin

## Slide 4
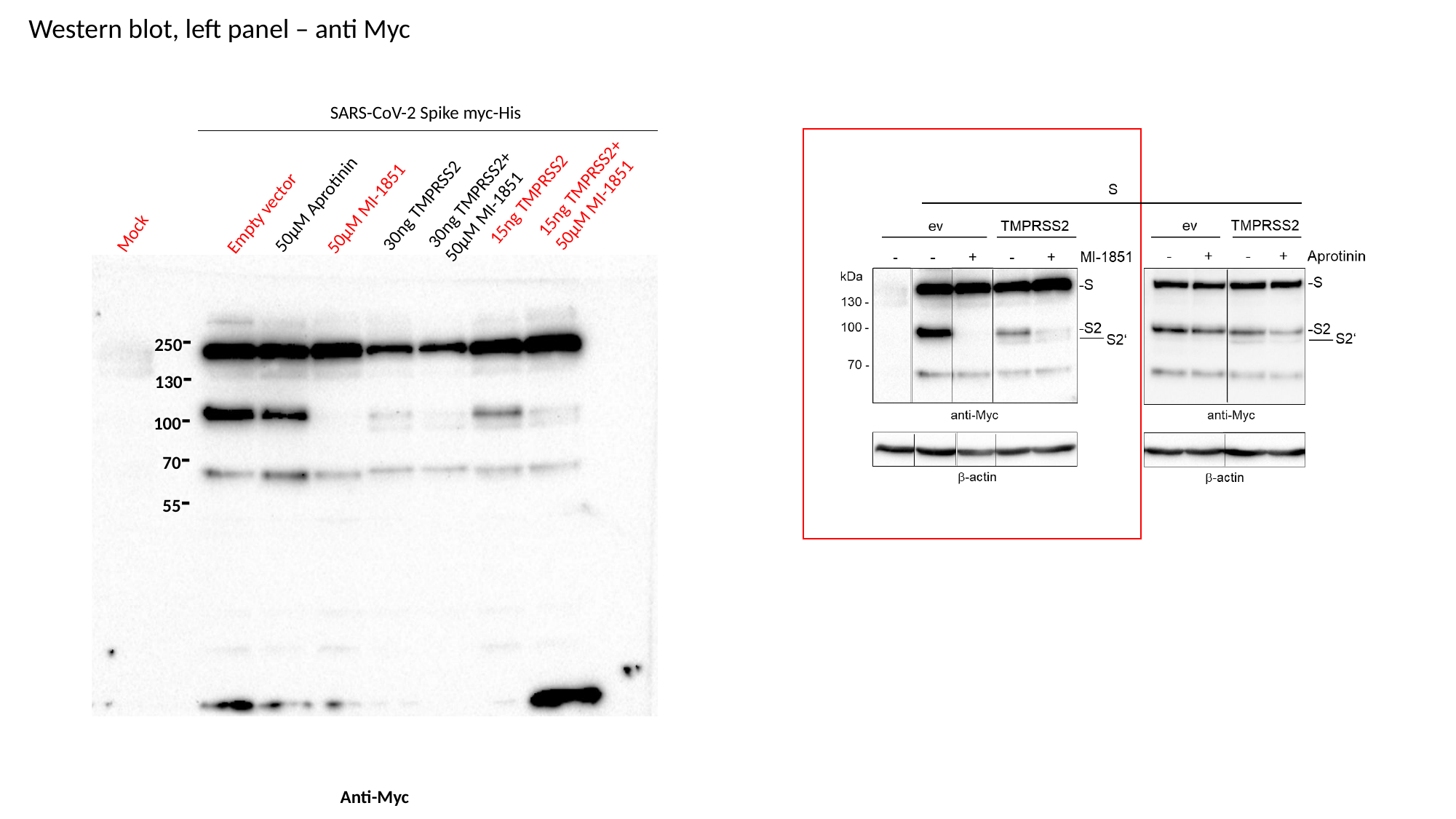

Western blot, left panel – anti Myc
SARS-CoV-2 Spike myc-His
15ng TMPRSS2+
50µM MI-1851
30ng TMPRSS2+
50µM MI-1851
15ng TMPRSS2
30ng TMPRSS2
50µM MI-1851
50µM Aprotinin
Empty vector
Mock
-S
-S2
S2‘
250-
130-
100-
70-
55-
Anti-Myc

## Slide 5
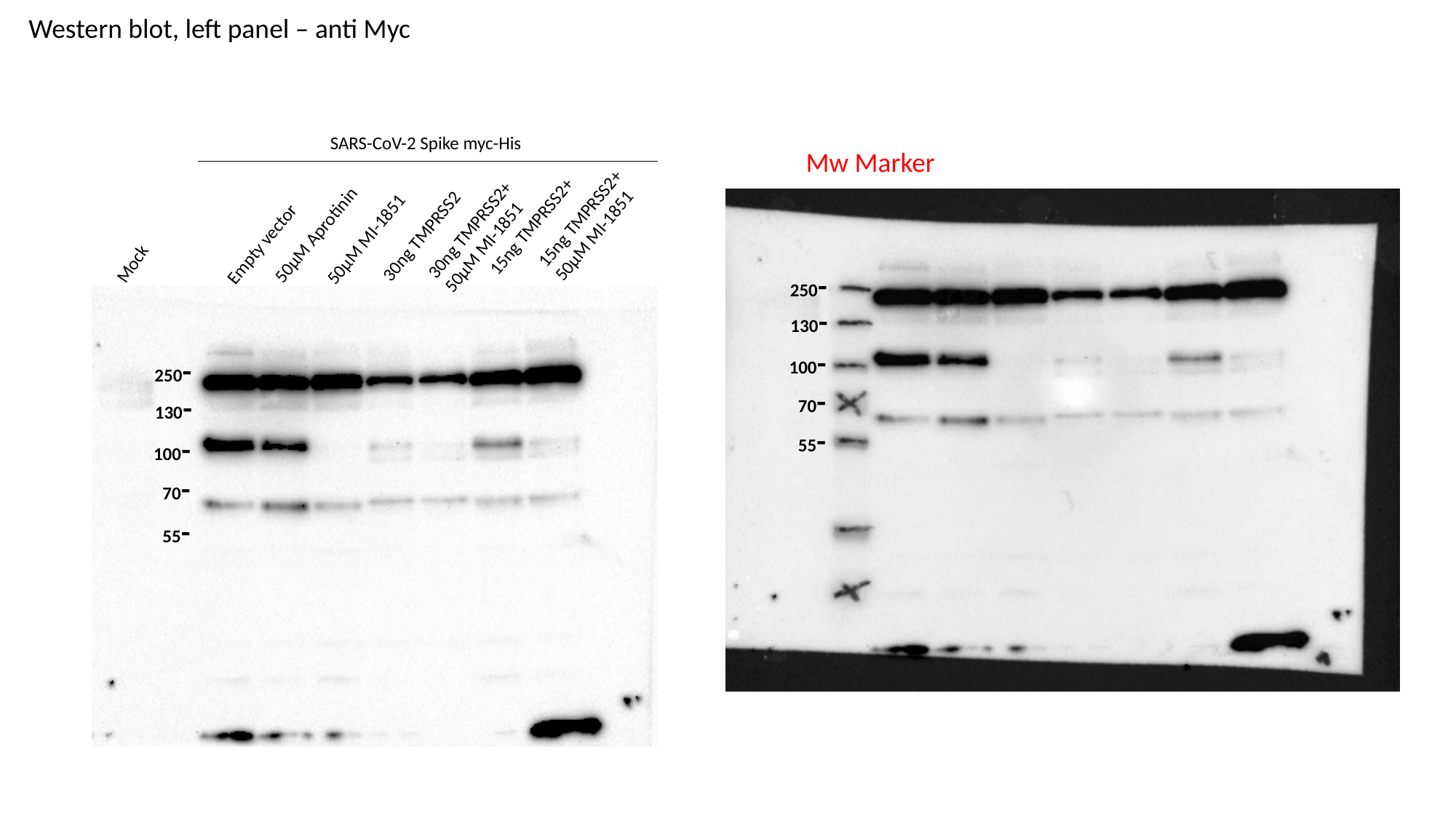

Western blot, left panel – anti Myc
SARS-CoV-2 Spike myc-His
Mw Marker
15ng TMPRSS2+
50µM MI-1851
30ng TMPRSS2+
50µM MI-1851
15ng TMPRSS2+
30ng TMPRSS2
50µM MI-1851
50µM Aprotinin
Empty vector
Mock
250-
-S
-S2
S2‘
130-
100-
250-
70-
130-
55-
100-
70-
55-

## Slide 6
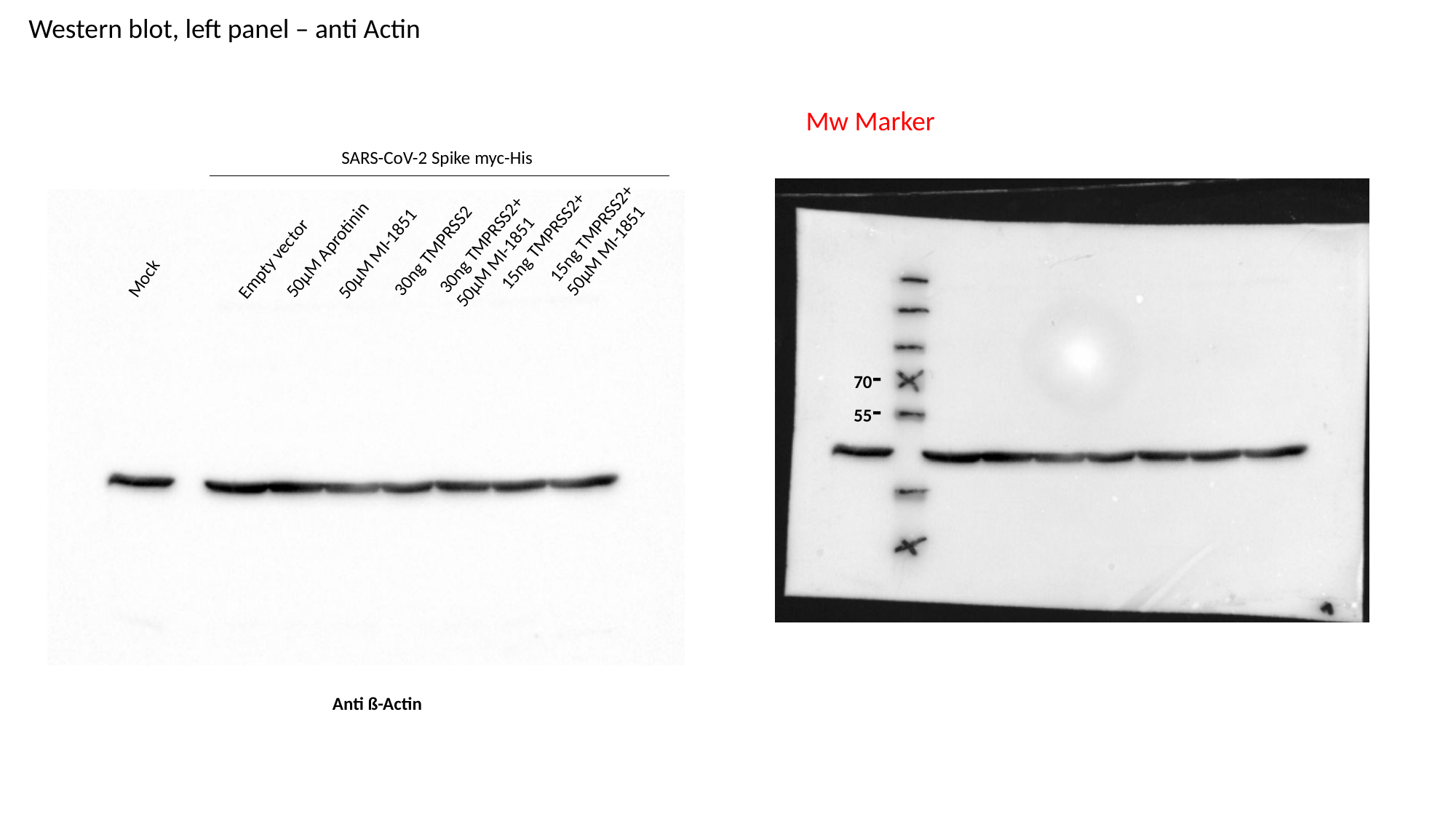

Western blot, left panel – anti Actin
Mw Marker
SARS-CoV-2 Spike myc-His
15ng TMPRSS2+
50µM MI-1851
30ng TMPRSS2+
50µM MI-1851
15ng TMPRSS2+
30ng TMPRSS2
50µM MI-1851
50µM Aprotinin
Empty vector
Mock
70-
70-
55-
55-
Anti ß-Actin

## Slide 7
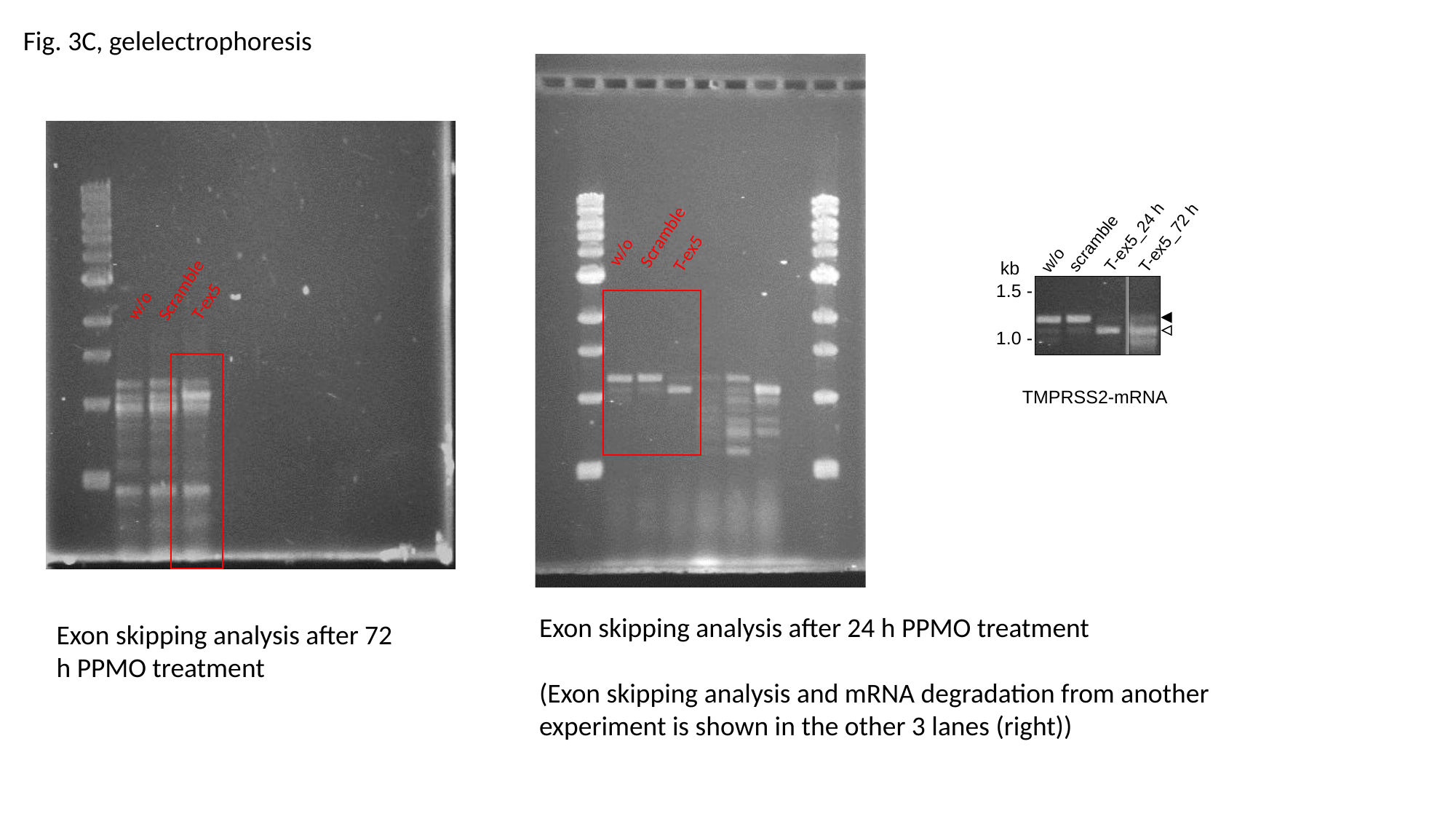

Fig. 3C, gelelectrophoresis
T-ex5_72 h
T-ex5_24 h
scramble
w/o
kb
1.5 -
1.0 -
TMPRSS2-mRNA
w/o
Scramble
T-ex5
w/o
T-ex5
Scramble
Exon skipping analysis after 24 h PPMO treatment
(Exon skipping analysis and mRNA degradation from another experiment is shown in the other 3 lanes (right))
Exon skipping analysis after 72 h PPMO treatment
